# Vitamin D enhances production of soluble ST2, inhibiting the action of IL-33

**DOI:** 10.1016/j.jaci.2014.09.044

**Published:** 2015-03

**Authors:** Paul E. Pfeffer, Yin-Huai Chen, Grzegorz Woszczek, Nick C. Matthews, Elfy Chevretton, Atul Gupta, Sejal Saglani, Andrew Bush, Chris Corrigan, David J. Cousins, Catherine M. Hawrylowicz

**Affiliations:** a MRC and Asthma UK Centre for Allergic Mechanisms of Asthma, King's College London, Guy's Hospital, London, United Kingdom; b Department of ENT, Guy's Hospital, Great Maze Pond, London, United Kingdom; c MRC and Asthma UK Centre for Allergic Mechanisms of Asthma, Imperial College London, Department of Paediatric Respiratory Medicine, Royal Brompton Hospital, London, United Kingdom; d Department of Infection, Immunity and Inflammation, University of Leicester, Glenfield Hospital, Leicester, United Kingdom

To the Editor:

Vitamin D insufficiency is an environmental factor that has been strongly associated with asthma and its severity.^1^ The genes *IL33* and *IL1RL1* have been repeatedly identified as predisposing to asthma risk in genome-wide association studies.^2^ IL-33 is an alarmin cytokine that acts on multiple pulmonary cell types, including T_H_2 lymphocytes, mast cells, and innate lymphoid cells, to promote T_H_2-type cytokine secretion and airway inflammatory responses of the kind observed in asthmatic patients.^3,4^ The receptor for IL-33 is encoded by *IL1RL1*; differential splicing of the gene can produce a functional membrane-bound receptor (ST2L) or a soluble decoy receptor (sST2).^5^ Therefore we investigated whether *IL1RL1* is regulated by vitamin D in cells relevant to asthma. We did this first by measuring gene expression by means of quantitative real-time PCR with 2 different TaqMan probe sets: Hs01073300, which detects mRNA splice variants encoding both the membrane-bound and soluble receptors (the total mRNA for *IL1RL1*), and Hs00249389, which detects mRNA only for the splice variant encoding the membrane-bound receptor ST2L (Fig 1, *A*). Methods are provided in this article's Online Repository at www.jacionline.org.

Both probe sets display similar efficiency of amplification (data not shown). Human primary bronchial epithelial cells (HBECs), CD4 lymphocytes, CD8 lymphocytes, eosinophils, and LUVA mast cells were cultured in the presence or absence of 1α,25-dihydroxyvitamin D3 (1,25[OH]D3), the active form of vitamin D (Fig 1, *B-D*, and see Fig E1, *A* and *B*, in this article's Online Repository at www.jacionline.org). Addition of 1,25(OH)D3 significantly increased the total number of *IL1RL1* mRNA transcripts expressed by HBECs and CD4 and CD8 lymphocytes, as measured by using the Hs01073300 probe set. However, 1,25(OH)D3 did not significantly increase expression of *IL1RL1* mRNA transcripts by primary eosinophils and LUVA mast cells, despite it significantly increasing expression of the gene cathelicidin antimicrobial peptide *(CAMP)*, which is known to be induced by vitamin D (see Fig E1, *A* and *B*).

Higher *IL1RL1* expression levels were detected with the Hs01073300 probe set than with the Hs00249389 probe set in HBEC and lymphocyte cultures. Although 1,25(OH)D3 increased the total copies of *IL1RL1* mRNA measured by using the Hs01073300 probe set in these cell types, it did not increase the number of transcripts encoding ST2L detected with Hs00249389, indicating that vitamin D selectively upregulates the expression of mRNA for the soluble decoy receptor sST2. sST2 concentrations in culture supernatants were measured by means of ELISA to confirm the findings of gene expression studies at the protein level. sST2 concentrations were significantly increased by 1,25(OH)D3 in both HBEC and CD4 lymphocyte cultures (Fig 1, *E* and *F*). In contrast, CD8 lymphocytes produced less than 200 pg/mL sST2 in all conditions, and there was no evidence of upregulation by 1,25(OH)D3 (data not shown).

Vitamin D circulates primarily as the inactive precursor 25-hydroxyvitamin D3 (25[OH]D3); however, epithelial cells constitutively express CYP27B1, the enzyme that converts 25(OH)D3 to 1,25(OH)D3.^6^ HBECs cultured with 100 nmol/L 25(OH)D3, a physiologic concentration, increased expression of *IL1RL1* (see Fig E1, *C*) and produced significantly greater amounts of sST2 (Fig 1, *G*). Primary human nasal epithelial cells (HNECs) cultured with a similar concentration range of 25(OH)D3 also showed a clear concentration-dependent increase in sST2 production (Fig 1, *H*). CD4 lymphocytes did not respond to 25(OH)D3, which is consistent with their lower expression of CYP27B1 (see Fig E1, *D*).

An IL-33 sensitive bioassay was developed using the LUVA mast cell line to test the biological activity of sST2. Twenty-four hours of exposure of LUVA cell cultures to IL-33 resulted in marked homotypic aggregation, which was associated with increased expression of CD54 (intercellular adhesion molecule 1; see Fig E2, *A* and *B*, in this article's Online Repository at www.jacionline.org). IL-33 increased CD54 expression on LUVA cells in a concentration-dependent manner (Fig 2, *A*). Other stimuli can also induce mast cell CD54 expression, and a similar concentration-dependent induction of CD54 expression was achieved with a combination of IFN-γ and IL-4 (Fig 2, *A*). Treatment of LUVA cells with a recombinant sST2-Fc chimera significantly inhibited IL-33–induced CD54 expression (*P* = .0076; Fig 2, *B*, and see Fig E2, *C*) but not IFN-γ plus IL-4–induced CD54 expression (see Fig E2, *D*). Addition of 1,25(OH)D3 itself to LUVA cell cultures did not significantly alter constitutive or induced CD54 expression (*P* > .05; see Fig E2, *E*), as previously reported for another mast cell line.^7^

To examine the effect of vitamin D–enhanced epithelial sST2 production on the IL-33 bioassay, HNECs were cultured in the presence or absence of 25(OH)D3 for 48 hours, and then the supernatants were added to cultures of LUVA cells stimulated with concentrations of IL-33 or IFN-γ plus IL-4 that induced comparable CD54 expression. Conditioned medium from vitamin D–treated epithelial cells significantly reduced IL-33–induced CD54 expression on LUVA cells compared with conditioned medium from matched epithelial cell cultures not treated with vitamin D (*P* = .015; Fig 2, *C* and *E*). In contrast, there was no significant difference in IFN-γ plus IL-4–induced CD54 expression between LUVA cells cultured with the different epithelial cell–conditioned media (*P* > .05; Fig 2, *D* and *E*). Although mediators in the epithelial cell–conditioned medium other than sST2 might conceivably have affected LUVA cell CD54 expression, the selective effect of vitamin D treatment on IL-33–induced CD54 expression suggests that the sST2 in the vitamin D–treated epithelial cell supernatants is biologically active.

The capacity of IL-33 to induce production of T_H_2-type proinflammatory cytokines by multiple cell types likely underpins the reported genetic associations of *IL33* and *IL1RL1* with asthma. Here we report the novel finding that 100 nmol/L vitamin D is able to augment expression by epithelial cells and lymphocytes of the soluble decoy receptor sST2, which in turn inhibits the actions of IL-33. Importantly, this effect occurs at physiologic concentrations (vitamin D sufficiency is defined as a serum 25[OH]D level of 75 to 150 nmol/L), and similar concentrations of 1,25(OH)D3 have been shown to be able to be generated from 25(OH)D3 in culture.^1^ We hypothesize that the capacity of vitamin D to augment the synthesis of an inhibitor of IL-33 *in situ* in the airways mucosa is of potential benefit in the limitation of asthmatic mucosal inflammation. Furthermore, this might in part account for the paradox that epidemiologic studies have repeatedly revealed associations between vitamin D insufficiency and both the risk of more severe asthma^1^ and increased serum IgE concentrations,^8^ whereas other studies have reported that in culture vitamin D can act directly on T_H_2 lymphocytes to promote T_H_2 cytokine secretion (eg, Boonstra et al^9^). The apparent beneficial association of vitamin D in asthma *in vivo*, which in many patients is a T_H_2-type cytokine pathology, suggests that the direct action of vitamin D in promoting T_H_2 lymphocyte responses is less important *in vivo* than other vitamin D–mediated mechanisms (eg, vitamin D upregulated production of sST2 and the induction of regulatory mechanisms) in inhibiting T_H_2-type cytokine responses. Because the enhancement by vitamin D of sST2 production is concentration dependent, this supports therapeutic strategies to boost pulmonary vitamin D levels to reduce asthmatic inflammation.

## Figures and Tables

**Fig E1:**
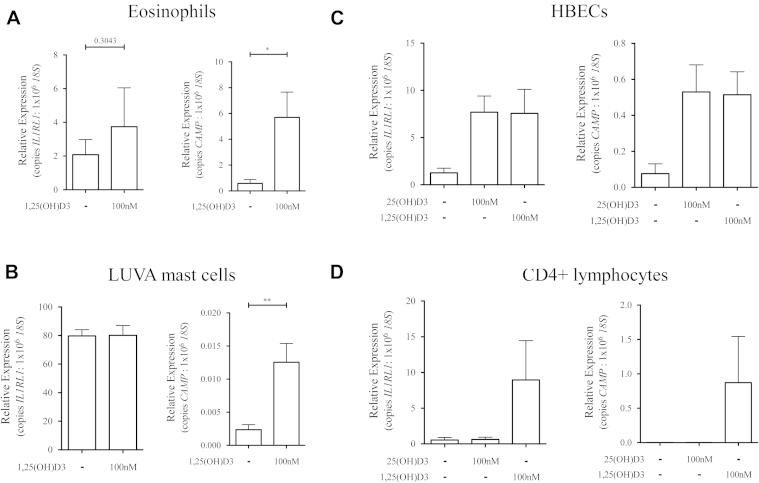
Further data on the effect of vitamin D on *IL1RL1* expression. **A** and **B,** 1,25(OH)D3 (100 nmol/L) increases the expression of mRNA for *CAMP* in primary human eosinophils (Fig E1, *A*) and LUVA mast cells (Fig E1, *B*) but not mRNA for *IL1RL1*, as measured by using the Hs01073300 probe set in either cell type (n = 5 for both cell types). **C** and **D,** 25(OH)D3 (100 nmol/L) increases the expression of mRNA for both *IL1RL1* and *CAMP* in HBECs (Fig E1, *C*) but not in CD4^+^ lymphocytes (Fig E1, *D*; Fig E1, *C*: n = 5; Fig E1, *D*: n = 3). *P ≤ .05 and **P ≤ .01.

**Fig E2:**
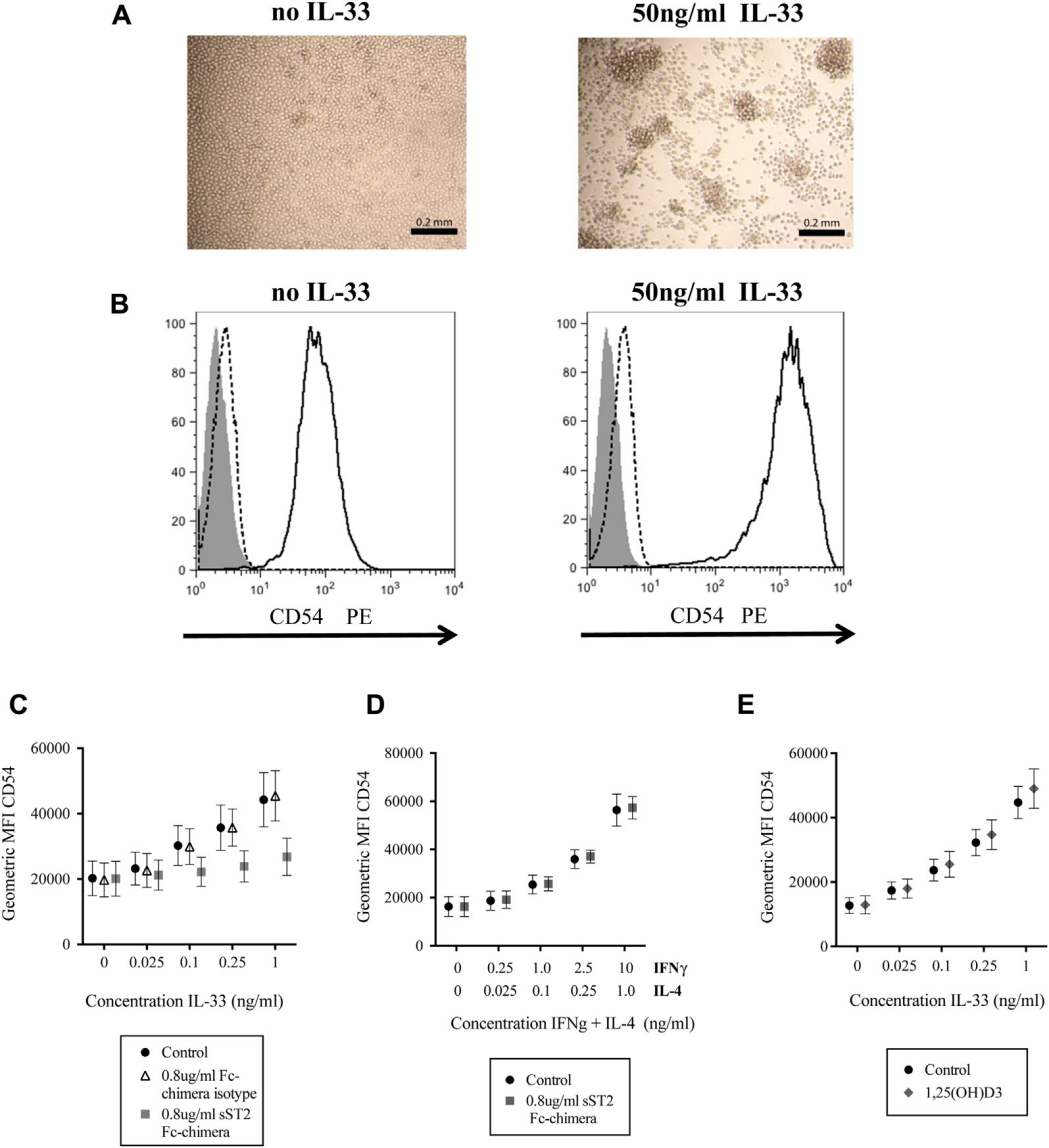
Stimulation with IL-33 induces LUVA cell expression of CD54. **A** and **B,** IL-33 causes homotypic aggregation of LUVA cells (×10 magnification; Fig E2, *A*) associated with increased expression of CD54 (*filled gray histogram*, unstained; *dashed line*, isotype control; *solid line*, anti-CD54; Fig E2, *B*). **C,** Inhibition of IL-33–induced LUVA cell CD54 expression by the recombinant sST2-Fc chimera is not observed with an isotype Fc chimera (n = 3). **D,** IFN-γ plus IL-4–induced LUVA cell CD54 expression is not inhibited by addition of recombinant sST2-Fc chimera (n = 4). **E,** Effect of IL-33 on LUVA cell CD54 expression is not affected by addition of active vitamin D (100 nmol/L 1,25[OH]D3) directly to the LUVA cells (n = 4).

**Fig 1:**
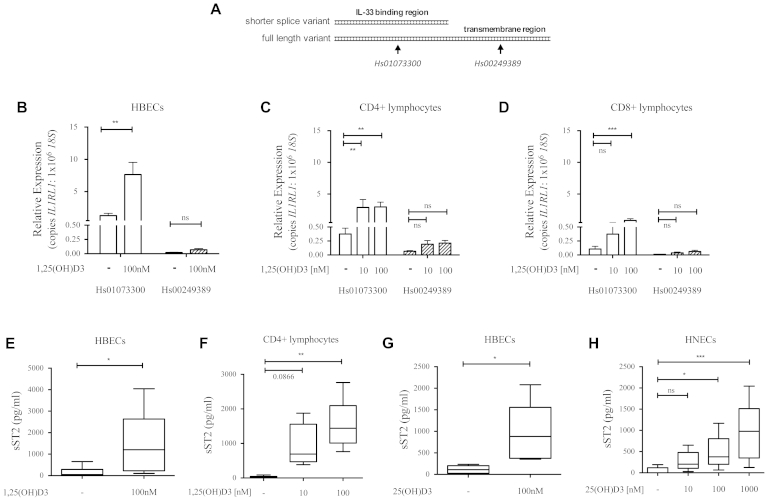
Vitamin D induces expression of sST2. **A,** Schematic for *IL1RL1* mRNA: the 2 splice variants are differentially detected in PCR by using the probe sets Hs01073300 and Hs00249389. **B-D,***IL1RL1* transcripts detected by each probe set in cultures of HBECs at 24 hours (Fig 1, *B*; n = 7) and CD4 (Fig 1, *C*) and CD8 (Fig 1, *D*) lymphocytes at 7 days (n = 6). **E-H,** sST2 protein in culture supernatants of HBECs at 24 hours (Fig 1, *E*; n = 6), CD4 lymphocytes at 7 days (Fig 1, *F*; n = 6), and HBECs (Fig 1, *G*; n = 6) and HNECs at 24 hours (Fig 1, *H*; n = 5). *ns*, Not significant. *P ≤ .05, **P ≤ .01, and ***P ≤ .001.

**Fig 2:**
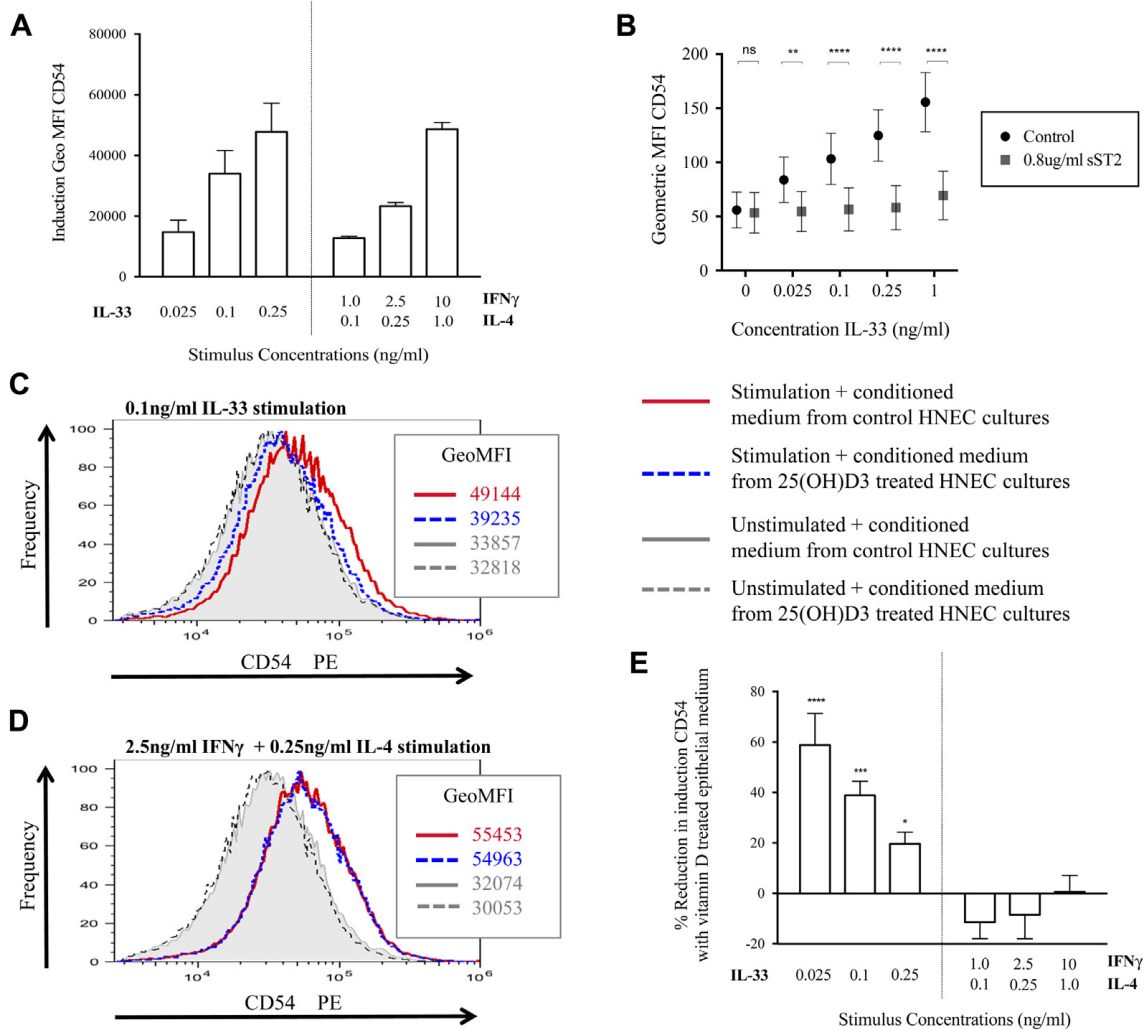
Conditioned medium from vitamin D–treated epithelial cultures inhibits IL-33–induced expression of CD54 by LUVA cells. **A,** Induction of CD54 on LUVA cells above that of unstimulated cells by different stimuli in the presence of conditioned medium from control epithelial cell cultures (n = 7). **B,** Effect of IL-33 on CD54 expression is inhibited by recombinant sST2 (n = 6). **C** and **D,** CD54 expression on LUVA cells cultured with conditioned medium with or without cell stimulation. **E,** Percentage reduction in stimuli-induced CD54 expression on LUVA cells cultured with conditioned medium from 25(OH)D3-treated epithelial cell cultures compared with control epithelial cell cultures (n = 7). *MFI*, Mean fluorescence intensity; *ns*, not significant. *P ≤ .05, **P ≤ .01, ***P ≤ .001, and ****P ≤ .0001.

## Methods

### Cell-culture reagents

Ultrahigh-purity 1,25(OH)D3 and 25(OH)D3 (Enzo Life Sciences, Exeter, United Kingdom) were placed in aliquots dissolved in dimethyl sulfoxide (Sigma-Aldrich, Gillingham, United Kingdom). Recombinant proteins were obtained as follows: IL-33, IFN-γ, IL-4, human sST2-Fc chimera, and isotype were all from R&D System (Abingdon, United Kingdom).

### Primary human epithelial cell culture

Primary HBECs were acquired from Lonza (Basel, Switzerland) and locally from endobronchial brushings obtained at fiberoptic bronchoscopy from adult volunteers (Guy's Research Ethics Committee, South London REC Office 3, REC approval no. 09/H0804/108, March 16, 2010). HNECs were obtained from resected nasal turbinates from adult patients undergoing nasal turbinectomy for clinical indications (City Road & Hampstead NRES Committee London, REC approval no. 12/LO/1931, January 14, 2013). Excised tissue was dissected and then treated with Liberase TL (Roche Diagnostics, Indianapolis, Ind) before isolation of epithelial cells with CD326 magnetic microbeads (magnetic cell sorting [MACS]; Miltenyi Biotech, Bisley, United Kingdom). Cells were expanded in flasks of Bronchial Epithelial Cell Growth Medium, which was constituted by supplementing Bronchial Epithelial Basal Medium (Lonza) with SingleQuot Supplements (Lonza). Epithelial cells were used for experiments at passages 3 to 5.

Cell purity after passage checked by means of flow cytometry with an allophycocyanin-labeled anti-human CD326 antibody (Miltenyi Biotech) and appropriate isotype control was greater than 99%.

Monolayers for cell-culture experiments were inspected daily by using light microscopy, and once approaching confluence, medium was changed to Bronchial Epithelial Cell Growth Medium containing all SingleQuot Supplements except bovine pituitary extract, retinoic acid, and hydrocortisone.

Cell-culture experiments with HBECs were conducted with triplicate wells for each condition in each experiment. For conditioned medium, culture supernatant was collected and centrifuged at 250*g* for 7 minutes before aliquots of the supernatant were used as conditioned medium.

### Peripheral blood lymphocyte culture

PBMCs were obtained by means of density centrifugation with Lymphoprep density gradient medium (Axis-Shield, Dundee, United Kingdom). CD4^+^ and CD8^+^ lymphocytes were isolated by using Dynabeads (Invitrogen, Life Technologies, Paisley, United Kingdom). Cell purity was assessed by means of flow cytometry with appropriate isotype controls and was 95% to 98% for all experiments. Lymphocytes were cultured at 1 × 10^6^ cells/mL in RPMI 1640 (Gibco, Life Technologies, Paisley, United Kingdom) supplemented with 50 U/mL IL-2 (Eurocetus, Harefield, United Kingdom), 10% FBS (PAA, Yeovil, United Kingdom), 2 mmol/L l-glutamine (Gibco, Life Technologies), and 0.1% 50 mg/mL gentamicin (Gibco, Life Technologies) on cell-culture plates coated with anti-CD3 (1 μg/mL; OKT-3) for 7 days.

### Peripheral blood eosinophil culture

PBMCs were obtained, as described previously. Eosinophils were isolated with a MACS negative selection kit (MACS, Miltenyi Biotech), according to an adapted manufacturer's protocol. Cell purity was 96% to 99% for all experiments. Eosinophils were cultured at 1 × 10^6^ cells/mL in RPMI 1640 supplemented with 10% FBS, 2 mmol/L l-glutamine, and 0.1% 50 mg/mL gentamicin for 20 hours.

### Quantitative real-time PCR

Epithelial cell monolayers were lysed with Qiazol reagent (Qiagen, Manchester, United Kingdom) before storage at −80°C pending extraction of mRNA with an miRNeasy Mini Kit (Qiagen), according to an adapted manufacturer's protocol with an off-column DNA digest with TurboDNase (Ambion, Life Technologies, Paisley, United Kingdom). mRNA was extracted from lymphocyte culture cell pellets by using an RNeasy Mini Kit (Qiagen), according to the manufacturer's protocol. mRNA was quantified with a NanoDrop ND-1000 Spectrophotometer (Thermo Scientific, Waltham, Mass) and converted to cDNA by using RevertAid Reverse Transcriptase and complementary reagents (Fermentas, Thermo Scientific). Relative quantification of *IL1RL1* relative to the *18S* housekeeping gene was conducted in triplicate by means of real-time quantitative PCR with the TaqMan primers Hs01073300 and Hs00249389 (Applied Biosystems, Life Technologies, Paisley, United Kingdom).

### sST2 ELISA

Concentrations of sST2 protein in cell-culture supernatants were quantified by using a Human ST2 ELISA DuoSet (R&D Systems), according to the manufacturer's protocol.

### LUVA cell culture and IL-33 bioassay

The LUVA mast cell line was a kind gift from Dr J. Steinke, University of Virginia.^E1^ LUVA cells were expanded in flasks in StemPro-34 serum free medium with associated nutrient supplement (Gibco, Life Technologies), l-glutamine (Gibco, Life Technologies), and 1% penicillin-streptomycin (10,000 U/mL, Gibco, Life Technologies). For the IL-33 bioassay, LUVA cells were cultured for 24 hours at 0.5 million cells/mL in RPMI 1640 supplemented with 10% FBS, 2 mmol/L l-glutamine, and gentamicin. The cell cultures were stimulated with a concentration series of recombinant IL-33 or IFN-γ plus IL-4 in the presence/absence of recombinant sST2-Fc chimera, isotype-Fc chimera, 100 nmol/L 1,25(OH)D3, or 40% HNEC conditioned medium. Cell-surface expression of CD54 (intercellular adhesion molecule 1) was measured by means of flow cytometry with a phycoerythrin-labeled anti-human CD54 antibody (BioLegend, London, United Kingdom) and appropriate isotype control on a FACSCalibur flow cytometer (BD Biosciences, San Jose, Calif) for initial experiments (Figs 2, *B*, and E2, *B*) and an Attune flow cytometer (Life Technologies, Paisley, United Kingdom) for later experiments (Fig 2, *A* and *C-E*, and Fig E2, *C-E*). Surface expression of CD54 was quantified as the geometric mean fluorescence intensity (GeoMFI). For stimulated LUVA cell cultures, induction of CD54 was calculated as the CD54 GeoMFI for unstimulated cell cultures subtracted from the GeoMFI for stimulated cell cultures in that experiment. The percentage reduction in stimulus-induced CD54 expression on LUVA cells cultured with vitamin D–treated epithelial cell–conditioned medium compared with induction of CD54 with control conditioned medium for each stimulus concentration was calculated to analyze the effect of vitamin D–treated epithelial cell–conditioned medium on stimulus-induced CD54 expression by LUVA cells.

### Statistical analysis

Data were analyzed with GraphPad Prism 6 software (GraphPad Software, La Jolla, Calif). For analysis of parametric data, *t* tests, repeated-measures ANOVA, and 2-way repeated-measures ANOVA were used with posttest-corrected multiple comparisons where the ANOVA was significant. For nonparametric data, Wilcoxon matched-pairs signed-rank tests or Friedman tests (with Dunn multiple comparison tests for comparison with control conditions) were used. Unless otherwise stated, parametric data are shown as means ± SEMs, and nonparametric data are shown as box and whisker plots with medians, 25th to 75th percentiles, and minimum to maximum ranges.
